# The Association Between Dental Caries and Salivary Buffering Capacity in Syrian Patients Diagnosed with Sickle Cell Disease

**DOI:** 10.7759/cureus.64887

**Published:** 2024-07-19

**Authors:** Lynn Ahmad, Abeer A Aljoujou, Reem Nadra, Ammar Mahmoud Mashlah, Fatima AlZahraa Al Beesh, Amr Alyafi, Haina Moulay Driss

**Affiliations:** 1 Department of Oral Medicine and Radiology, University of Damascus, Faculty of Dentistry, Damascus, SYR; 2 Department of Biology, University of Damascus, Faculty of Dentistry, Damascus, SYR; 3 Department of Dentistry, University of Damascus, Faculty of Dentistry, Damascus, SYR

**Keywords:** hydroxyurea impact, salivary buffer capacity, decayed missing and filled teeth (dmft), hbss, sickle cell disease complications

## Abstract

Background

Sickle cell disease (SCD) is a genetic disorder caused by mutations in the *HBB* gene, resulting in the abnormal shape of red blood cells. This condition is accompanied by various oral manifestations including salivary gland dysfunction leading to a heightened susceptibility to dental caries. This disorder is primarily treated with hydroxyurea. This study aims to assess dental caries utilizing the decay, missing, filling teeth (DMFT) index and evaluate salivary buffering capacity in patients diagnosed with SCD (HbSS type). The study also aims to assess the relationship between DMFT and salivary buffering capacity. Additionally, the study aimed to find a correlation between treatment with hydroxyurea and changes in both dental caries and salivary buffering capacity.

Methods

This case-control study enrolled a total of 100 participants aged between 20 and 50 years. The participants were divided into two groups: the study group, which comprised 70 individuals diagnosed with SCD (HbSS type), who were asked to report their current use of hydroxyurea, and the control group, which included 30 healthy individuals. Dental caries were assessed using the DMFT index, while salivary buffering capacity was measured using a pH meter model 420A device.

Results

The study group exhibited a mean DMFT index value of 6.39 compared to 5.20 in the control group. This difference was statistically significant (*P-*value=0.037), indicating higher DMFT values among patients with SCD. Salivary buffering capacity was significantly lower in the study group compared to the control group, with average values of 6.47 and 6.88, (*P-*value=.022). Interestingly, the administration of hydroxyurea impacted salivary buffering capacity, resulting in lower values for individuals using the drug (*P-*value=0.039). Conversely, hydroxyurea did not have a significant effect on DMFT values (*P-*value=0.317).

Conclusion

SCD increases susceptibility to dental caries and is associated with significant changes in salivary composition. At the same time, the potential negative impact of hydroxyurea is acknowledged.

## Introduction

Sickle cell disease (SCD) is a prevalent genetic disorder characterized by mutations in the *HBB *gene responsible for the beta-globin chain formation [1, 2]. These mutations result in the sickle-shaped alteration of red blood cells. The inheritance of this genetic mutation can occur either homozygously or heterozygously, with sickle cell anemia specifically referring to the homozygous condition. Other types of SCD include HbSC (sickle cell hemoglobinopathy C) and sickle cell beta Thalassemia [3]. The primary feature of SCD is pain crisis resulting from vaso-occlusion, which leads to tissue blood flow restriction and subsequent tissue death [4]. This disease affects various body systems, including the oral cavity, necessitating appropriate healthcare provision for individuals with SCD [5]. All patients experience varying degrees of chronic hemolytic anemia and vascular obstruction, along with damage to body organs [6], and oral manifestations commonly include oral mucosa pallor due to chronic anemia [7]. Pulp necrosis can occur independently of dental caries or injuries due to blood vessel obstruction in the pulp [8-10]. Neuropathy may present in the trigeminal nerve, with a particular predilection for the mental branch of the inferior alveolar branch, causing numbness or a burning sensation along the nerve pathway [7, 8, 11]. Ulcers on the oral mucosa may also develop due to anemia and fungal infections following multiple antibiotic treatments [11]. Furthermore, SCD can lead to salivary gland dysfunction, attributed to the accumulation and adherence of sickle-shaped red blood cells in the small blood vessels of the salivary glands, consequently impacting saliva quality and affecting oral health [12]. Thus, patients with SCD are at a higher risk for dental caries, largely attributable to these salivary changes [13].

Saliva plays a crucial role in the formation of dental caries due to its buffering capacity, which is a vital factor in preventing caries. However, research on the influence of oral health on the quality of life of patients is limited. A study by Medeiros et al. in 2018 revealed a high prevalence of caries and salivary changes in individuals with SCD, rendering them especially susceptible to dental caries [13, 14]. Saliva is a complex fluid that affects oral health through its physical and chemical properties. Disruptions in the quality or quantity of saliva can have adverse effects on oral health [15]. Saliva is predominantly composed of 99% water, with the remaining 0.5% consisting of various organic and inorganic substances, as well as cellular elements. It plays a pivotal role in digestion by providing moisture and aiding in the mastication of food [4, 16]. The buffering capacity of saliva is widely regarded as one of the most reliable indicators of caries susceptibility [17]. Bicarbonate, as the primary buffering agent, counteracts acids and shields the teeth from acid exposure from dietary sources. By resisting pH alterations caused by acidic or basic substances, salivary buffering helps maintain a stable pH and acts as a defense against dental caries [18-20].

Hydroxyurea is the primary therapeutic approach and the only available treatment for the majority of individuals with SCD. Also known as hydroxycarbamide, it is an approved safe and effective treatment method that promotes the production of fetal hemoglobin (HbF). Hydroxyurea has been authorized by the US Food and Drug Administration since 1998 and the European Medicines Agency since 2007 for its efficacy in managing SCD. This drug significantly reduces the incidence of vaso-occlusive crisis, hospitalizations, and mortality rates associated with SCD [21-23]. Other medications such as L-glutamine, crizanlizumab, and voxelotor are also used as adjuvant agents or second-line treatments in the management of SCD [23]. Blood transfusion can also enhance blood flow within blood vessels by reducing the number of circulating sickle red blood cells, thereby serving as a preventive measure against vaso-occlusive attacks and averting vascular occlusion [21].

SCD is widespread in Syria, with an estimated 2,500 infected individuals in 2009 and approximately 200 new cases expected annually [24]. Given the prevalence of SCD in Syria and its various impacts on the oral cavity, one significant effect is the potential obstruction in the microvasculature of the salivary glands. This obstruction can disrupt the salivary glands’ functions, leading to an adverse impact on the development of dental caries and overall oral health [12].

The purpose of this study is to assess dental caries using the decayed, missing, filled teeth (DMFT) index and evaluate salivary buffering capacity in a sample of Syrian patients with SCD. Additionally, the study aims to explore the relationship between the DMFT index and salivary buffering capacity and investigate the effects of hydroxyurea on the DMFT index and salivary buffering capacity in the study group.

## Materials and methods

Participants and study design

**Figure 1:**
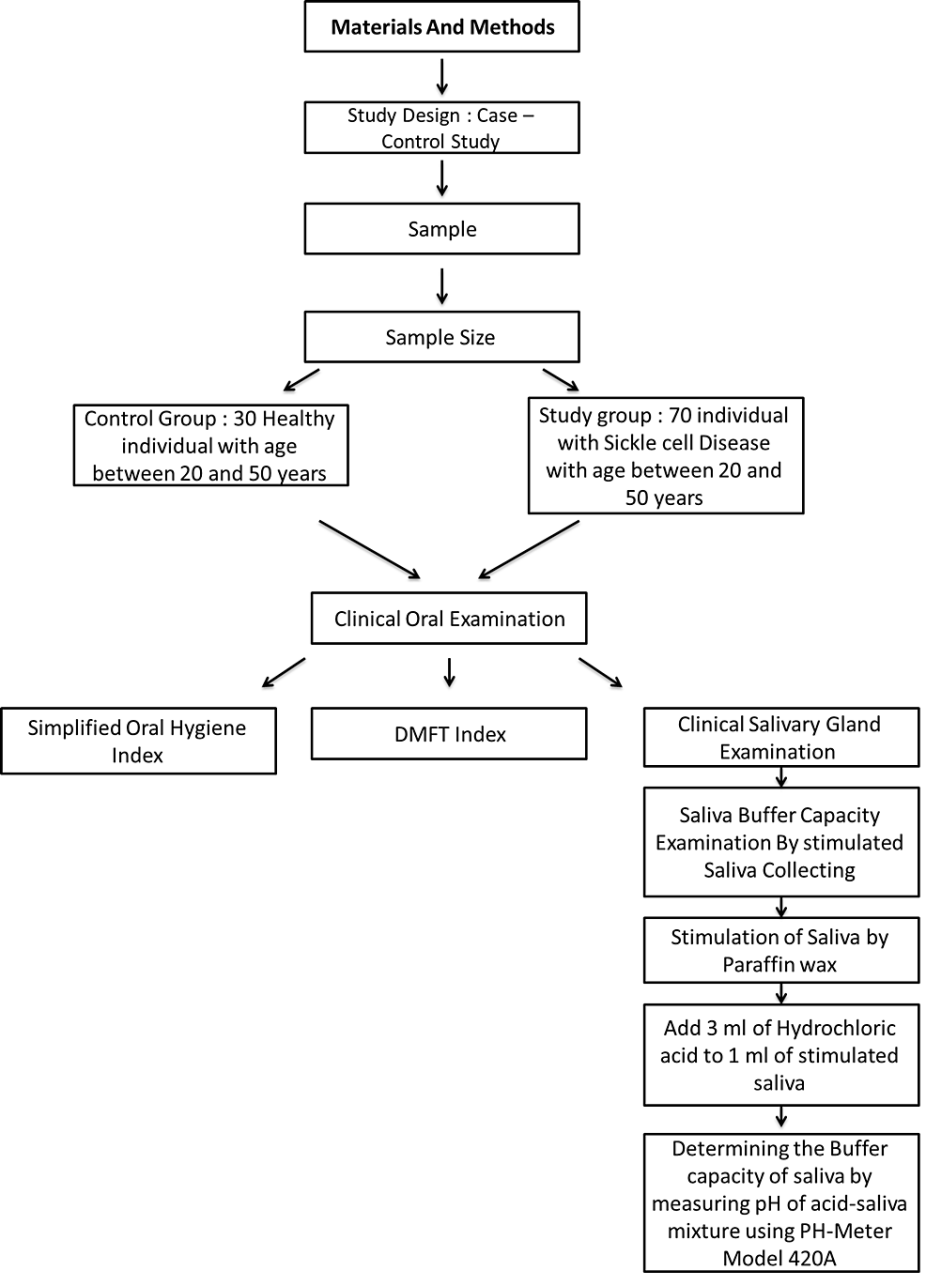
Methodology The figure is drawn by the authors of this article.

This case-control study was conducted between December 2022 and April 2023 at three locations: The Comprehensive Clinics Center - Department of Genetic Hematology, the Department of Oral Medicine at the Faculty of Dentistry, and the Faculty of Pharmacy at the University of Damascus. The study included a total of 100 individuals aged between 20 and 50 years, divided into two groups: a study group (SCD group) that comprised 70 individuals diagnosed with SCD (HbSS type). Among the 70 participants in the SCD group, 63 individuals underwent hydroxyurea treatment, while the remaining seven participants did not receive such treatment. Additionally, a control group comprised 30 healthy individuals. Participants with SCD who were smokers, alcoholic, pregnant, undergoing orthodontic treatment, or had other systemic diseases were excluded from the study group. Similarly, Individuals from the control group diagnosed with systemic diseases or using any medication were excluded (Figure 1).

Clinical oral examination and the studied parameters

**Figure 2:**
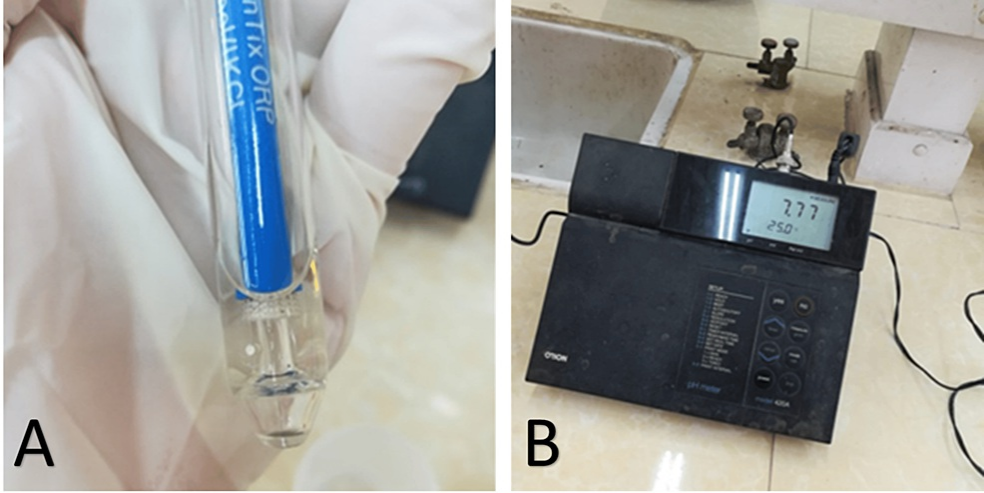
pH meter model 420A device utilized in the study. A) The glass electrode submersed in the saliva sample, B) the electronic meter that displays the corresponding pH value.

An extensive dental and salivary gland examination was conducted to assess the oral health status of each patient. The DMFT index and simplified oral hygiene index (OHI-S) were documented [25]. Furthermore, stimulated saliva samples were collected from the patients to determine the buffering capacity. Saliva secretion was initiated using graduated plastic containers and paraffin wax. Upon obtaining the stimulated saliva sample, 3 ml of 0.005% hydrochloric acid was added to 1 ml of saliva. Subsequently, the pH meter 420A device was utilized to measure and display the pH value of the acid-saliva mixture (Figure 2). A pH level value above 6.0 was considered normal, while a pH value of 5.5 indicates low buffering capacity, and a pH value below 4 indicates very low buffering capacity [14]. Prior to the salivary tests, patients were instructed not to eat or drink for at least an hour, refrain from brushing their teeth one hour before the test, maintain a comfortable posture, and avoid stress during the examination. All salivary tests were conducted between 9:00 and 11:00 in the morning. In addition, patients were required to complete a regarding their hydroxyurea treatment status. Following each clinical examination, patients received detailed guidance on oral health and care.

Sample size

The sample size for this study was determined using sample size calculator proportion, with a significant level set at 0.05 and confidence level at 0.95, based on previous studies conducted by Davidopoulou et al. in 2021 and AL-Alawi et al. in 2015 [4,26,27].

Statistical analysis

The student’s t-test was used to examine the statistical relationship between median values for DMFT and buffering capacity values in both the study group and the control group. Additionally, it aimed to determine the correlation between treatment with hydroxyurea and changes in the DMFT index and salivary buffering capacity. The Pearson correlation coefficient was employed to investigate the relationship between DMFT and salivary buffering capacity, results were considered significant if the P value was less than 0.05.

## Results

A total of 100 individuals, aged between 20 and 50 years, participated in this study. The sample was divided into two groups: the study group, consisting of 70 individuals diagnosed with SCD (HbSS type), with 63 of these individuals undergoing a therapeutic regimen involving hydroxyurea, and the control group, which included 30 healthy individuals selected based on matched characteristics of age and sex.

The SCD group showed higher mean DMFT index values (6.39) compared to the control group (5.20), signifying a statistically significant difference (P value=0.037), indicating an elevated high risk of dental caries among SCD patients. In contrast, the mean salivary buffering capacity values were lower in the SCD group (6.47) than in the control group (6.88), with a statistically significant difference (P value=0.022), suggesting reduced salivary capability to prevent dental caries in SCD patients (Table 1). These findings indicate that the consistent oral care level across all patients highlights the substantial impact of diminished salivary buffering capacity on the heightened occurrence of dental caries.

**Table 1:** The effect of sickle cell disease on DMFT index and salivary buffering capacity. DMFT: decayed, missing, filled teeth *Statistically significant

	Group	Number	Mean	Standard Deviation	Standard Error	Minimum	Maximum	Student’s t-Test	P-value
DMFT index	SCD	70	6.39	2.68	0.32	3	19	2.114	0.037^*^
	Control	30	5.20	2.30	0.42	2	15		
Salivary buffering capacity	SCD	70	6.47	0.61	0.07	4.09	6.86	-2.369	0.022^*^
	Control	30	6.88	0.42	0.08	5.19	7.78		

Additionally, a statistically significant correlation was observed between DMFT index values and salivary buffering capacity values specifically within the SCD group (P value=0.004), while no correlation was found in the control group (P value=0.056), indicating that compromised salivary buffering capacity in SCD patients heightens the incidence of dental caries (Table 2).

**Table 2:** The correlation between DMFT index and salivary buffering capacity. DMFT: decayed, missing, filled teeth *Statistically significant

	Group	Number	Pearson Correlation	P-value
Correlation between DMFT index and salivary buffering capacity	SCD	70	-0.343	0.04^*^
	Control	30	-0.352	0.056

In the SCD group, all patients were deemed at high risk of dental caries, regardless of their use of hydroxyurea. However, the use of hydroxyurea led to a variance in buffering capacity values, manifesting lower values in patients receiving the drug (P value=0.039). Nevertheless, hydroxyurea did not show a significant variation in DMFT index values (P value=0.317), implying that its usage inhibits salivary buffering capacity, thereby clinically increasing dental caries occurrence, despite the lack of statistical correlation between hydroxyurea use and dental caries escalation (Table 3).

**Table 3:** The effect of hydroxyurea on DMFT index and salivary buffering capacity. DMFT: decayed, missing, filled teeth *Statistically significant

	Group	Hydroxyurea	Number	Mean	Standard Deviation	Standard Error	Minimum	Maximum	Student's t-Test	P value
DMFT index	SCD	Yes	63	6.48	2.70	0.34	3	19	-1.007	0.317
		No	7	5.33	2.42	0.99	3	11		
Salivary buffering capacity	SCD	Yes	63	6.02	0.62	0.08	4.09	6.86	2.110	0.039^*^
		No	7	6.56	0.24	0.10	6.11	6.74		

## Discussion

SCD is a globally recognized health issue that raises concerns about its impact on oral health due to various changes it induces in oral tissues [8,14]. This study specifically focused on patients with SCD (HbSS type), which is one of the most prevalent hereditary diseases in Syrian society. The disease significantly affects oral health in all its aspects, necessitating preventive programs that prioritize oral and dental health. Dental infection and its complications, if left untreated, can trigger or exacerbate vaso-occlusive crisis [9,28,29].

Effect of sickle cell disease on DMFT

Factors contributing to the occurrence of dental caries in SCD patients include infrequent dental visits, resulting in inadequate healthcare provision, and frequent hospital admissions due to disease-related complications, leading to neglect of oral health [9,28]. Dentists often display reluctance in treating SCD patients due to concerns over potential complications arising from the disease [30]. Additionally, SCD patients may experience a reduction in salivary flow rate, further increasing the risk of dental caries development, particularly in cases of oral health neglect [31]. The findings of this study align with previous studies conducted by Al-Alawi et al. in 2015, Brandão et al. in 2018, Davidopoulou et al. in 2021, and Laurence et al. in 2006, [4,14,26,32]. However, there are discrepancies with the study by Fernandes et al. in 2015, as the average DMFT index values in SCD children were lower than that of the control group. The researcher attributes this discrepancy to the provision of free dental care and the increased attention to healthcare for these children by their families. Conversely, there was no difference in the average DMFT index values between the study and control groups in adolescents. The researcher interprets this finding as a consequence of adolescents seeking more independence, leading to a reduced focus on preventive measures and oral care [9]. Furthermore, our study disagrees with the findings of Passos et al. in 2012, which showed no statistically significant difference in DMFT index values between the study and control groups. This discrepancy may be attributed to the inclusion of two types of SCD, HbSS (the most severe type) and HbSC (less severe), in their sample, whereas our study focused solely on the HbSS type [30]. Lastly, our study diverges from the findings of Matos et al. in 2014, which found no statistically significant difference in the average DMFT/dmft index values between the study and control groups. This result may be attributed to an absence of variation in sugar intake frequency and frequent tooth brushing among members of both groups [33].

Effect of sickle cell disease on salivary buffering capacity

There are three distinct buffer systems found in saliva, namely the protein buffer, phosphate buffer, and bicarbonate buffer. Among these, the bicarbonate buffer plays the most crucial role and is primarily activated in stimulated saliva [19]. While there is limited research on the impact of SCD on salivary buffering capacity, the available results are inconclusive. It is postulated that the reduced glomerular filtration rate commonly observed in adults with SCD could be a potential cause for low buffering capacity values in these patients [34]. This is due to impaired kidney function leading to decreased absorption of bicarbonate, which is the primary salivary buffer, and reduced hydrogen excretion in urine, consequently resulting in diminished bicarbonate concentration in saliva and subsequently diminished buffer capacity [35]. In our study, we observed statistically significant differences in buffering capacity values, with lower values detected in the SCD group compared to the control group. This finding is consistent with the findings reported by Brandão et al. in 2018, who also observed lower buffering capacity values among SCD patients [14]. However, our results contradict the findings of de Matos et al. in 2014, who reported significantly lower buffering capacity compared to the SCD group [33].

Variation in DMFT index after hydroxyurea treatment

The current study did not find any significant effect of the hydroxyurea drug on the DMFT index. Only 10% of individuals in the SCD patient group did not take the drug, compared to 90% who did. Even though, all patients exhibiting low salivary buffering capacity values were receiving hydroxyurea therapy. It is noteworthy, however, that the drug’s common renal side effects could potentially impact salivary buffering capacity. This differs from the study by Salvia et al. in 2013, which found higher DMFT index values in 30 patients taking the drug compared to 39 patients not taking it [36]. Another study by Brandão et al. in 2018 showed that 33 adolescents taking the drug had lower DMFT values compared to 28 adolescents not taking it. In terms of primary teeth, dmft values were higher among those taking the medication. The number of children taking the medication was 14, compared to 9 who were not taking it [14]. The difference in findings may be attributed to variations in the number of individuals taking the drug and those not taking it in different studies.

Variation in salivary buffering capacity after hydroxyurea treatment

Hydroxyurea may cause a decrease in salivary buffering capacity due to its potential to cause dry mouth [14]. Our study found that individuals taking the drug had lower salivary buffering capacity values compared to those not taking it. This finding aligns with the studies conducted by Brandão et al. in 2018 and Salvia et al. in 2013, which also reported low salivary buffering capacity in individuals taking the drug [14,36].

Correlation between DMFT values and salivary buffering capacity

Dental caries is a complex disease influenced by various factors [37]. In our study, it was observed that saliva exerts a protective effect against dental caries owing to its buffering capacity. A significant inverse correlation was identified between DMFT values and salivary buffering capacity within the study group. Specifically, higher values of salivary buffering capacity were associated with lower DMFT values, indicating a potential protective relationship against dental caries [38]. However, no significant correlation was found between salivary buffering capacity and DMFT values in the control group. This is consistent with the study by Medeiros et al. in 2018 but differs from the findings of Brandão et al. in 2018, which reported higher DMFT values in the SCD group with normal salivary buffering capacity [13,14]. The variance in DMFT values between the study groups may be attributed to the multifactorial nature of dental caries.

This study has several limitations that warrant consideration. Challenges associated with sample collection from individuals undergoing pain crises, the exclusion of patients with concurrent systemic diseases, and the significant exclusion of smokers from the study are notable limitations. Additionally, the potential influence of psychological factors on salivary flow rate constitutes a further confounding variable. These limitations impede the generalizability of the findings to broader populations and should be taken into account when interpreting the results. Therefore, it is advisable to undertake future research endeavors that address these limitations in order to gain a more comprehensive understanding of the influence of hydroxyurea on salivary characteristics and oral health in individuals with SCD.

## Conclusions

SCD is a common monogenic disease worldwide, and dental infections can trigger painful attacks in patients. Evaluating the oral health of SCD patients is crucial, as SCD elevates the risk of dental caries and causes an alteration in salivary composition. Hydroxyurea is the primary treatment for SCD and helps reduce symptoms of the disease. However, it may have a negative effect on saliva, and further studies may help elucidate the impact of hydroxyurea on saliva and the oral health of SCD patients.
